# Molecular dynamics study of low molecular weight gel forming salt-triggered dipeptide

**DOI:** 10.1038/s41598-023-33166-3

**Published:** 2023-04-18

**Authors:** Xiangfeng Jia, Jingfei Chen, Wen Xu, Qi Wang, Xiaofeng Wei, Yongshan Ma, Feiyong Chen, Guiqin Zhang

**Affiliations:** 1 School of Municipal and Environmental Engineering, Shandong Jianzhu University, Jinan, 250101 Shandong China; 2 Key Laboratory Biofuels and Shandong Provincial Key Laboratory of Synthetic Biology, Qingdao Institute of Bioenergy and Bioprocess Technology, Chinese Academy of Sciences, Qingdao, 266101 China; 3 Institute of Resources and Environment Innovation, Shandong Jianzhu University, Jinan, 250101 Shandong China

**Keywords:** Chemistry, Materials science

## Abstract

Molecular dynamics simulation method was used to study the aggregation of Na and Ca salts in different concentrations of Naphthalene-dipeptide (2NapFF) solutions. The results show that high-valence Ca^2+^ triggers the formation of a gel at a certain dipeptide concentration, and the low-valence Na^+^ system follows the aggregation law of general surfactants. The results also show that hydrophobic and electrostatic forces are the main driving forces for the formation of dipeptide aggregates, and that hydrogen bonds do not play a major role in the formation of dipeptide solution aggregates. Hydrophobic and electrostatic effects are the main driving forces for the formation of gels in dipeptide solutions triggered by Ca^2+^. Electrostatic attraction drives Ca^2+^ to form a weak coordination with four oxygen atoms on two carboxyl groups, which causes the dipeptide molecules to form a gel with a branched network structure.

## Introduction

Low molecular weight gels (LMWGs) are the major types of network hydrogels and organogels, and are a class of soft materials that have attracted substantial interest during the past decades because LMWGs with easily attainable and modifiable chemical structures can spontaneously self-assemble to form 3D networks and immobilize the liquid phase^1–4^. LMWGs have been extensively explored in many fields, such as drug delivery^5,6^, cancer therapy^7^, catalysts^8^, bio-materials^9^, chirality and energy transfer^10^, sensors^11^, pollution scavengers^12^, as well as in recent three-dimensional^13^ and four-dimensional printing material-processing techniques^14^.

Among the different types of LMWGs, peptide-based hydrogels belong to one of the best-represented classes of gels because of their good biocompatibility, reversibility, and biodegradability. They are widely used in tissue engineering^15^, cell culture^16^, and drug delivery, as antimicrobial coatings^17^. Functionalized dipeptides can be effective LMWGs^18–21^. These hydrogels are typically formed through a number of ways, most commonly by a temperature^22,23^, pH^24,25^, a suitable salt^18,19,26^, or by an enzymatic reaction on a precursor to the gel^27^.

Therefore, the formation mechanism of this type of gel is a research hotspot. The gelators self-assemble into supramolecular structures, including fibers, tapes, and tubes; gelation is a result of the entanglement or cross-linking of such structures. Adams^22^ states that it is inappropriate to say that self-assembly leads to gelation, but rather self-assembly leads to the formation of structures that, under appropriate conditions, interact to form a network that results in the formation of a gel. Much of our current understanding is derived from indirect experimental measurements. However, experimental techniques cannot adequately explain the molecular arrangement of LMWGs within gel fibers, junction zones, and the aggregate-solvent interfaces^28^.

To understand the stability and aggregation behavior of molecular gels, molecular-level information, such as the aggregate size distribution, aggregation stability, binding modes, and roles of different functional groups of LMWGs, is required. Molecular simulations have provided an effective way to understand the mechanical properties of self-aggregation and the relationship between the molecular interactions and functional properties of a hydrogel^29,30^. The all-atom (AA) molecular dynamics simulations have emerged as a unique tool for providing insight into the aggregation phase during supramolecular gelation^31^.

Naphthalene-dipeptide (2NapFF) ^18^, a typical example of salt-triggered gels, has been reported in the experiments at an alkaline pH, most often above pH 9^32,33^. The apparent pKa of the terminal carboxylic acid of 2NapFF is equal to 6.^34^ At high pH, the carboxylic acid is deprotonated and carboxylate is formed in the dipeptide solutions; hence, the dipeptide is seen as a surfactant^18,35,36^. Solutions of dipeptides that formed worm-like micelles most often resulted in the formation of gels when a Ca salt was added^18,19,32,37^. Therefore, worm-like micelles must be present for Ca-triggered gelation. 2NapFF forms gels at high pH; however, in experiments, it is still uncertain if the structure is crucial or whether there are further structural changes.

To further understand the formation mechanism of LMWGs formed by salt-triggered peptide molecules, molecular dynamics were used in this study to simulate the aggregation process of 2NapFF surfactant solutions (Na carboxylate or Ca carboxylate) of different concentrations at high pH. Through the analysis of the aggregate microstructure, the structure and intermolecular interactions of the gels were explained at the microscopic level and the aggregation mechanism was explored and studied.

**Figure 1:**
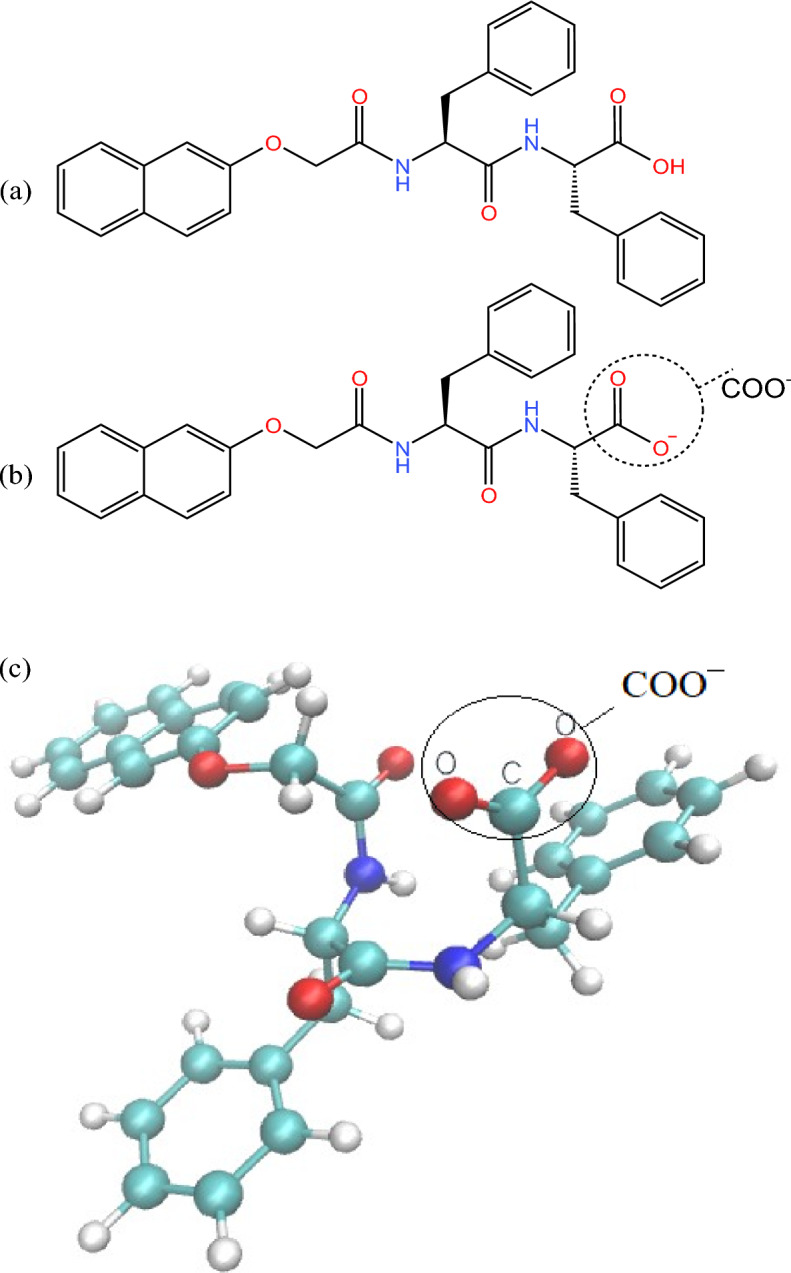
Structure of 2NapFF. (**a**) the molecule of 2NapFF; (**b**) at high pH, the deprotonated structure with a negative charge; (**c**) the ball and stick model (CPK) of the deprotonated structure.

The molecular structure of 2NapFF is shown in Fig. 1, where (a) is the molecular structure, (b) is the deprotonated structure with a negative charge at high pH, and the carboxylate (COO^−^) is marked in Fig. 1b,c. Figure 1c shows the ball-and-stick model (CPK) of the deprotonated structure in the molecular graphics software VMD^38^, which is also the structural model used in the subsequent simulation process.

## Results and discussions

### The structure of the aggravations of all the simulation systems

**Figure 2:**
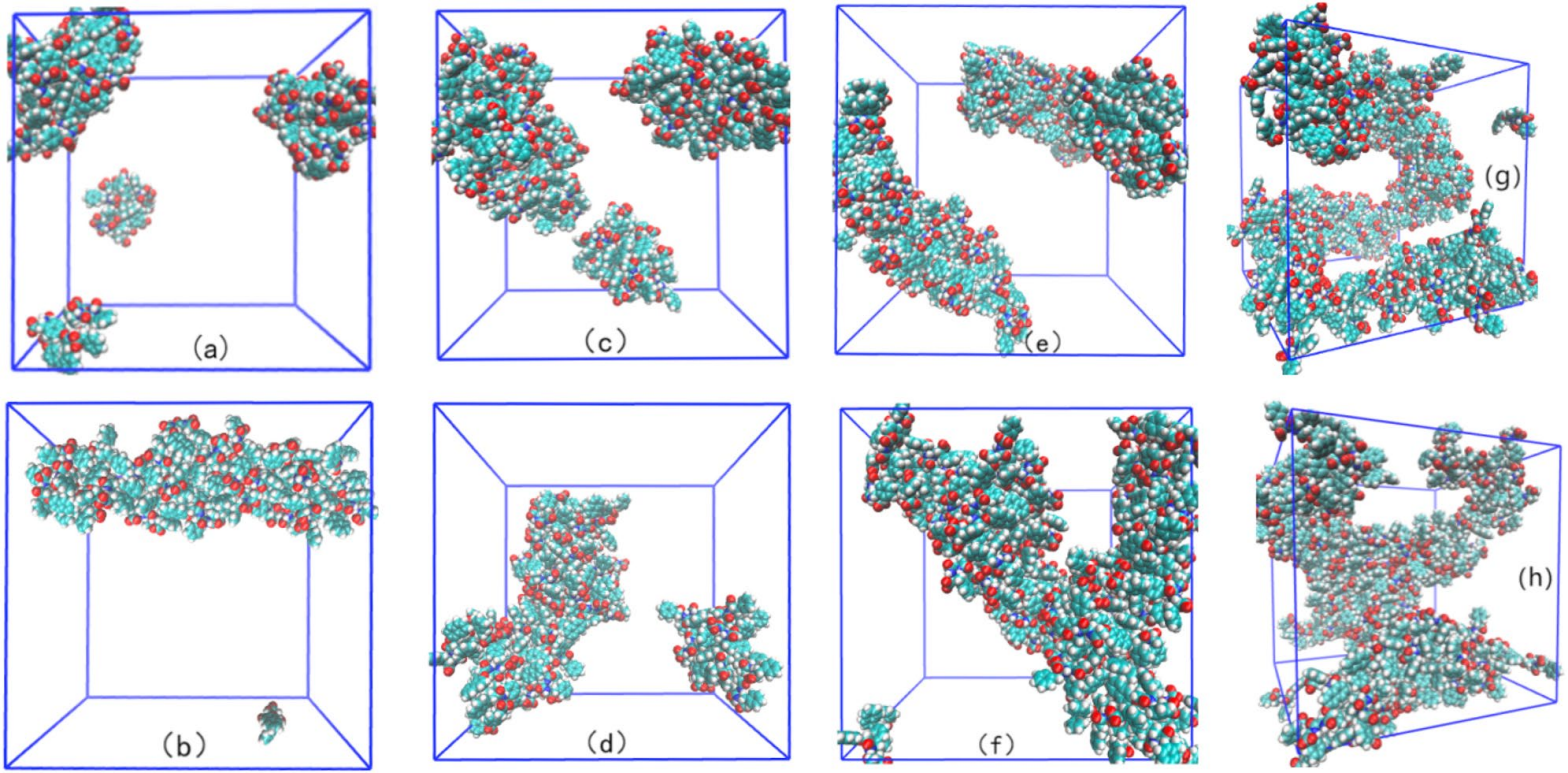
The stages of aggregate formation in the systems. Marks (a-h) correspond to systems (1–8) in Table 1. Water and ions are removed from the images.

Figure 2 shows the final equilibrium aggregate states corresponding to the eight simulation systems listed in Table 1. For clarity, the water and ions in the simulation cell were removed from all the images. In Fig. 2, the top row is the Na carboxylate system, followed by Ca carboxylate systems with the same concentration of 2NapFF as described above, and the concentrations of 2NapFF increase gradually from left to right.

**Table 1:** The detailed compositions and the final aggregate states.

System	water	2NapFF	Na^+^	Ca^2+^	Cl^−^	The final aggregate state
(1)	14,000	40	40	0	0	Spherical micelle
(2)	14,000	40	0	20	0	Rod(worm)-like micelle
(3)	14,000	80	80	0	0	Spherical(rod-like) micelle
(4)	14,000	80	0	40	0	Rod(worm)-like micelle
(5)	14,000	120	120	0	0	Worm-like micelle
(6)	14,000	120	0	60	0	Cross-linking worm micelle (gel)
(7)	14,000	160	160	0	0	Long worm-like micelle
(8)	14,000	160	0	80	0	Cross-linking worm micelle(gel)

The stable aggregation states of the systems after equilibrium show that with the increase in 2NapFF concentration, the aggregates of the odd (Na^+^ triggered) systems change from the initial spherical micelles to rod (worm)-like micelles, and then to the final long worm-like micelle, which is consistent with the aggregation properties of general surfactants. After replacing Na^+^ with Ca^2+^ corresponding to the same concentration of 2NapFF, the aggregation capacity becomes intense, followed by the formation of rod-like micelles at the beginning of the Ca^2+^ salt systems. When system (5) of Na^+^ salt forms worm-like micelles, corresponding to the same concentration of the Ca^2+^ salt system (6), it forms a branched structure. When the Na^+^ salt system (7) forms a long worm-like structure, it corresponds to a more complex branch network structure (gel) triggered by Ca^2+^ ions in system (8) with the same concentration of 2NapFF.

In the description of the experimental results, when adding Ca salt to induce gel formation, gels are formed where a worm-like micelle phase is present^18,19,24,41^. This implies that there are structural transformations when the Ca salt is added and the gelation is not simply a result of “locking in” the micellar structures. This finding is consistent with our observations. Comparing the aggregates of systems (5), (6), (7), and (8), in our simulation, it can be seen that the aggregates induced by Na^+^ ions tend to form worm-like micelles in a long direction, while the aggregates triggered by Ca^2+^ tend to branch to a three-dimensional network structure and form the gel, especially the branched network more obvious in system (8). This shows that the Na^+^-triggered dipeptides form worm-like micelles and the addition of Ca^2+^ to the original worm-like micelles leads to the formation of a gel with a network structure. This gelation is the result of structural transformation that occurred after reaching a certain concentration (above the concentration formed by worm-like micelles).

Therefore, to better understand the role of salt ions in the formation of gels, the following analysis focuses on the four systems (5, 6, 7, 8) with worm-like micelles and gel formation.

### The hydrogen bond interaction between 2NapFF

According to Hamachi et al., the presence of a large number of hydrogen bonds in the hydrogel system is the main driving force for gelation^42,43^. Hydrogen bonds are defined to satisfy the following conditions:^44^

$${\text{R}}_{{{\text{DA}}}} \le 3.5\mathop {\text{A}}\limits^{ \circ } {, }\quad \, {\text{and}}\quad \phi \le 30^{^\circ }$$

here *R*_AD_ is the distance between the hydrogen bond donor and acceptor and *Ф* is the angle of H—D…A.

**Figure 3:**
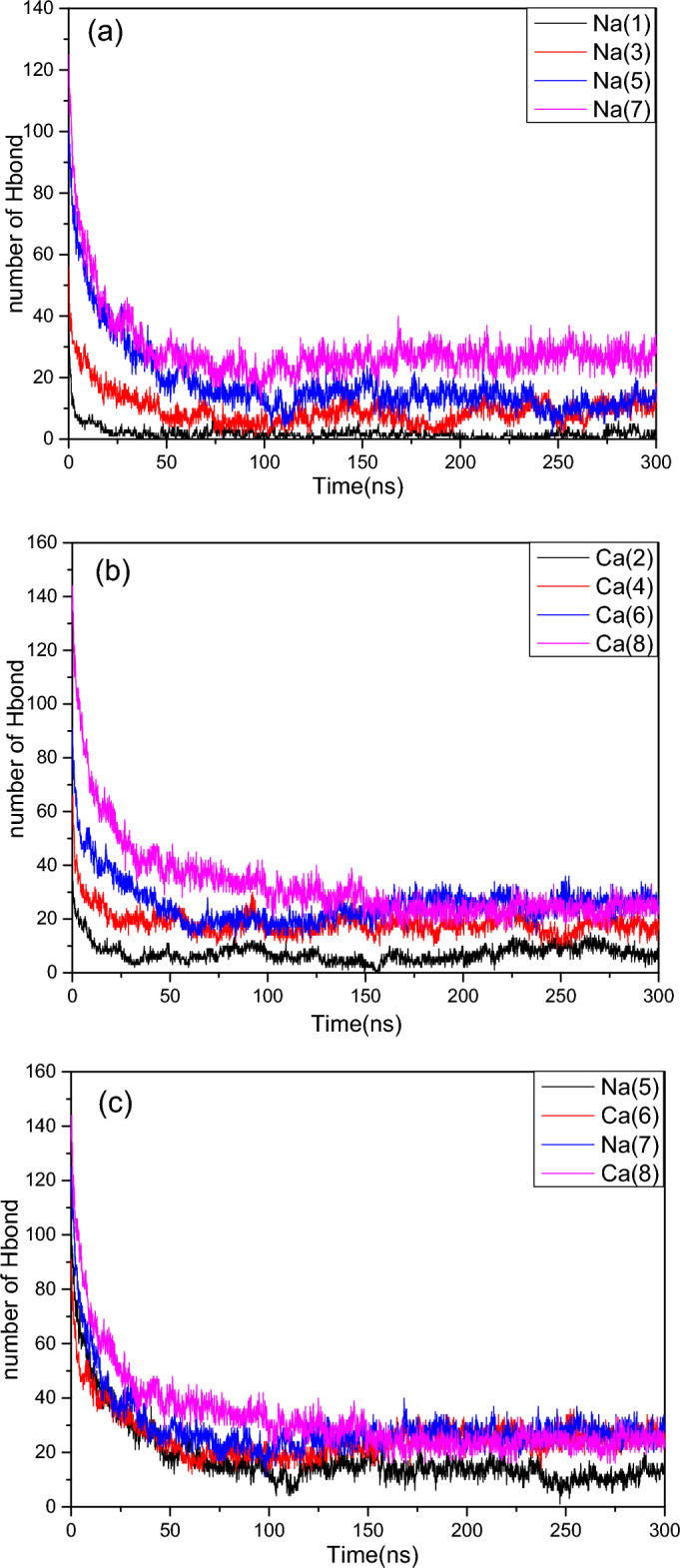
The number of hydrogen bonds between the dipeptides (2NapFF). (**a**) Na carboxylate system (1, 3, 5, 7); (**b**) Ca carboxylate system (2, 4, 6, 8); (**c**) the Ca^2+^ system(6, 8) forming gel corresponds the Na^+^ system(5, 7) with the same dipeptides concentration.

Figure 3 shows the number of hydrogen bonds formed between the dipeptides (2NapFF) corresponding to the different systems in Table 1. As shown in Fig. 3, the number of hydrogen bonds in all simulation systems gradually stabilized after 30 ns, that is, the systems gradually formed stable aggregates. After 150 ns, the number of hydrogen bonds in all systems remained unchanged, indicating the formation of the final stable aggregations. In Fig. 3a, the number of hydrogen bonds in Na^+^ systems (1, 3, 5, 7) is given; with increasing concentrations of dipeptides, the number of hydrogen bonds increases gradually. Figure 3b shows the numbers of hydrogen bonds in Ca^2+^ systems (2, 4, 6, 8); the number of hydrogen bonds also increase with the increasing of concentration of dipeptides at the beginning. After the formation of stable aggregates (approximately 150 ns), the number of hydrogen bonds in the systems of (6, 8) formed in the gels tended to be equal. Figure 3c shows the number of hydrogen bonds in the systems (5–8), which shows that the numbers of hydrogen bonds in gel systems (6) and (8) are primarily the same as those in the long worm-like micelle formation system (7). After all the systems were stable, the number of hydrogen bonds was small, even though the number of hydrogen bonds in the gel system (8) with a clear network structure was slightly lower than that of the long worm-like system (7) formed at the same dipeptide concentration. This indicates that hydrogen bonds are not the main factor in the formation of salt-ion-triggered dipeptide gels.

### The radial distribution calculation (rdf)

Inter- and intra-molecular interactions were calculated by radial distribution calculation (rdf), the distance between the groups was measured, and the peak height was proportional to the probability that the interacting groups would be at a certain distance from each other.

**Figure 4:**
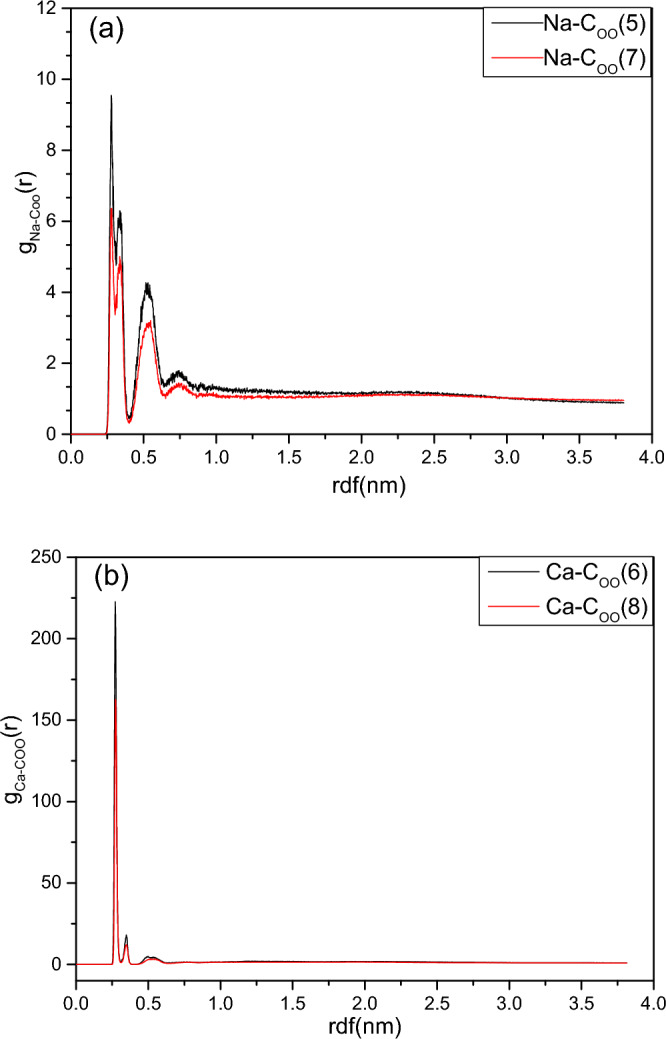
Radial distribution function (RDF) g_Na-COO_(r) of (**a**) Na^+^-C_OO_ in systems (5, 7); (**b**) Ca^2+^-C_OO_ in systems (6, 8).

Figure 4 shows the rdf between the metal salt ions and carbon atoms of the carboxylic (C_OO_) in 2NapFF for the simulation systems (5, 6, 7, 8). Figure 4a shows the rdf (g_Na-COO_(r)) between Na^+^ and C_OO_ in systems (5,7), and Fig. 4b shows the rdf (g_Ca-COO_(r)) between Ca^2+^ and C_OO_ in systems (6, 8). Figure 4a shows that the distance distribution between Na^+^ and carboxylate in systems (5) and (7) is consistent, most Na^+^ distribute at 0.25–0.55 nm from carboxylate and a small amount is also distributed at 0.7–0.8 nm. Figure 4b shows that the distribution of Ca^2+^ in systems (6) and (8) is also consistent, with a very strong main peak within 0.3 nm. This indicated that the distribution of Ca^2+^ was more regular than that Na^+^. Almost all Ca^2+^ was distributed near the carboxylate, except for a small amount of discrete Ca^2+^.

**Figure 5:**
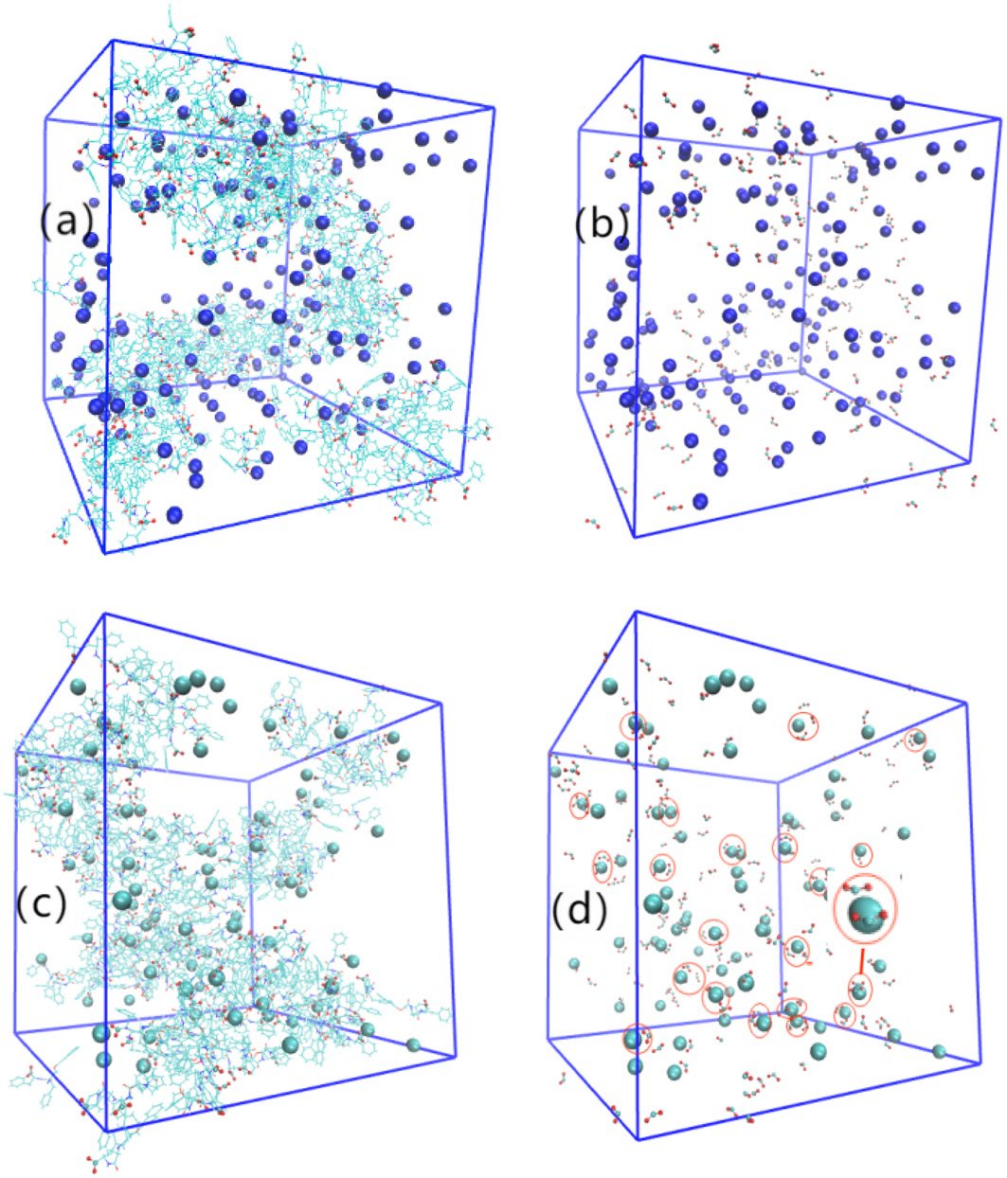
The images of the long worm-like micelle (7) and gel in system (8). (**a**): Only Na^+^ and 2NapFF; (**b**) only Na^+^ and carboxylate COO^−^; (**c**) only Ca^2+^ and 2NapFF; (**d**) only Ca^2+^ and carboxylate COO^−^.

To better illustrate the distribution of metal salt ions in the aggregates, Fig. 5 provides images of Na^+^, Ca^2+^, carboxylic acids, and the acid groups in systems (7) and (8). As shown in Fig. 5b, except for Na^+^ ions that were distributed around the long worm-like micelle, other ions were randomly distributed in the simulation cell, while Ca^2+^ ions were distributed in the outer layer of the gels in Fig. 4b. The distributions of Na^+^ and Ca^2+^ in Fig. 5 are consistent with the radial distribution function shown in Fig. 4.

As shown in Fig. 5a,b, most of the carboxyl groups of the dipeptides face outward, and the negatively charged carboxyl groups can pair with positively charged Na^+^. One Ca^2+^ with two positive charges and two carboxyl groups are usually distributed around it and paired with it, as shown in Fig. 5c,d. In the anions of carboxylates, owing to the delocalization of electrons, their two-carbon oxygen bonds are actually the same; therefore, four oxygen atoms in two carboxyl groups have the same attractions as Ca^2+^.

**Figure 6:**
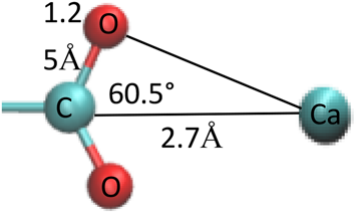
The geometric diagram of Ca^2+^ closed to the carboxyl group of 2NapFF.

From the detailed data file of the hydrogen bond and Fig. 4b, it can be seen that the main peak of g_Ca-COO_(r) was approximately 0.27–0.28 nm. Figure 6 shows the geometry of the carboxyl group of a 2NapFF molecule and Ca^2+^. The CO bond length of the COO^−^ group was approximately 1.25 Å and the O–C–Ca angle was approximately 60.5°, half of the O–C–O angle (121°)^45^. According to the geometric cosine theorem (c^2^ = a^2^ + b^2^ − 2abcosθ), the O–Ca bond length is calculated approximately 2.35–2.53 Å , which is equivalent to the coordination bond formed by the physical association of Ca^2+^ and the COO^−^ group of the amino acid^45–47^.

A large number of studies on the coordination of Ca^2+^ in aqueous solutions have shown that although the position of Ca^2+^ and the surrounding coordination group are different, the most common coordination number around Ca^2+^ is between 7–8^47–50^. The theoretical research^46^ on the interaction between amino acids and Ca^2+^ in aqueous solution shows that Ca^2+^ forms 7–8 weak bond coordination compounds with the carboxyl groups of amino acids and water molecules. For example, when Ca^2+^ coordinates two oxygen atoms of the carboxyl group on the main chain of phenylalanine and the oxygen atoms of six surrounding water particles, the saturated eight-coordination configuration is the most stable.

Therefore, we speculate that when Ca^2+^ triggers dipeptides to form a gel, it should form a stable coordination configuration with four O atoms on two carboxyl groups and oxygen on the surrounding water molecules; thus, the distribution of Ca^2+^ is very regular.

Owing to the three-dimensional coordination configuration of Ca^2+^, the two carboxylates of the dipeptides coordinated with it freely adjust in the angle and direction. Thus, it is easier for dipeptide molecules to form branched network structures and trigger the formation of gels, rather than forming long aggregates in a one-dimensional direction. Compared with Ca^2+^, Na^+^ only carries a positive charge and has poor coordination ability; therefore, the aggregation law follows the aggregation mechanism of general surfactants that aggregate from spherical micelles to rod (worm-like) micelles, and then to long worm-like micelles.

### The Lennard–Jones potentials and the Coulomb potentials

**Figure 7:**
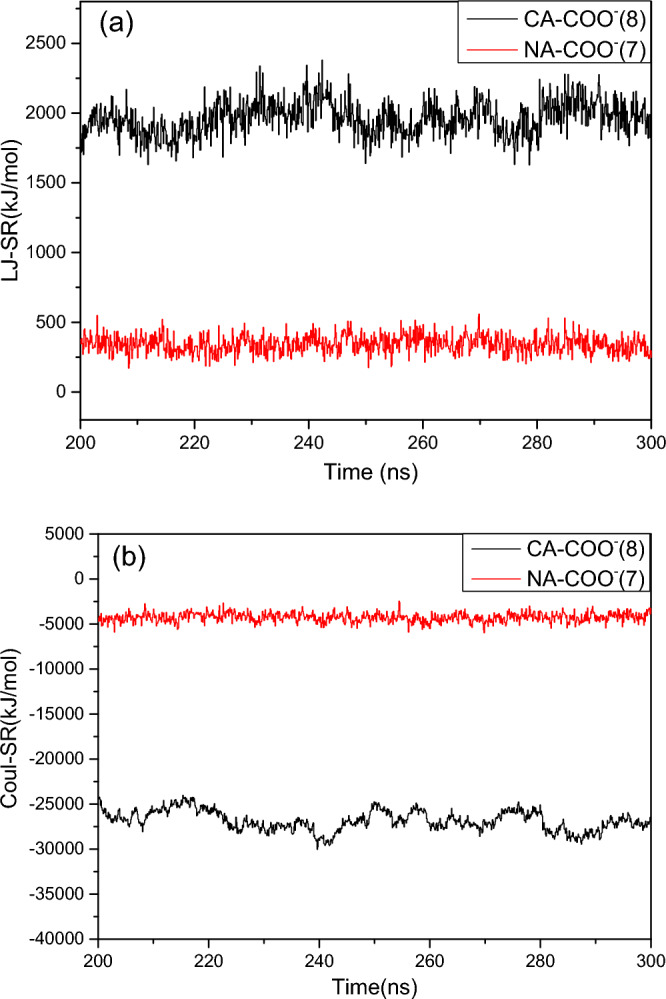
Interaction as revealed by potentials for the systems (7) and (8) from 200–300 ns. (**a**) The short range LJ potential (LJ-SR) of ions (Ca^2+^, Na^+^) and the carboxylate COO^−^; (**b**) the short range coulomb potential (Coul-SR) ions (Ca^2+^, Na^+^) and the carboxylate COO^−^.

The electrostatic effect is an important factor in aggregate formation. To better understand the process of positively charged ions in the formation of gel and long worm-like micelles, the van der Waals interaction (LJ potential) and electrostatic interaction energy (Coulomb potential) in the simulation processes of systems (7) and (8) should be calculated. The non-bonded short-range Lennard–Jones (LJ-SR) potential and the Columbic short range potential (Coul-SR) are calculated, here, the COO^−^ group of the 2NapFF molecules is defined separately in the structure file, and Fig. 7 shows the LJ-SR and Coul-SR potentials between the COO^−^ group and Ca^2+^ or Na^+^ in the last 100 ns (from 200 to 300 ns) after the aggregates are stabilized in systems (7) and (8).

Figure 7a shows the LJ-SR potential of the COO^−^ group and Na^+^ and Ca^2+^ with the same concentration of 2NapFF; it can be seen that the van der Waals interaction of the COO^−^ group and Ca^2+^ in system (8) is higher than that of Na^+^ and COO^−^ groups in system (7). Figure 7b shows the Coul-SR potential in systems (7) and (8) for the COO^−^ group and Na^+^ in system (7) and Ca^2+^ (8). The negative sign represents the attraction between positively charged ions and the negatively charged COO^−^ group, and the Coul-SR potential between Ca^2+^ and COO^−^ group is much stronger than that between Na^+^ and COO^−^ group. It is evident in Fig. 7 that the LJ-SR and Coul-SR potentials in system (8) are stronger than those in system (7).

Figure 7a,b show that the Coul-SR potential representing the electrostatic interaction between COO^−^ groups and ions (Na^+^, Ca^2+^) is an order of magnitude higher than the LJ-SR potential. This indicates that electrostatic potential energy plays an important role in the formation of salt induced aggregates (including micelles and gels). This also proves that the strong electrostatic attraction caused by the charge of Ca^2+^, that is, the electrostatic potential, plays a more important role in gel formation.

### The solvent accessible surface area (sasa) of 2NapFF groups

The protonated dipeptide molecule has a hydrophilic head group and a hydrophobic long chain; thus, the hydrophobic effect on the formation of aggregates requires attention. However, it is difficult to accurately calculate the hydrophobic force. In order to understand the importance of hydrophobic forces in the formation of gel by dipeptide (2NapFF), and the solvent-accessible surface area (sasa) of the dipeptide group can be calculated after forming stable aggregates. The accessible surface area of the solvent was calculated using Gromacs software, which is usually a water molecule with a radius of 1.4 Å. When calculating the Sasa, three parts are mainly calculated: the total area of the selection group (the dipeptide group is selected here), the hydrophobic area, and the hydrophilic area of the selection group. The hydrophobic and hydrophilic areas were distinguished based on the atomic charges in the selection group molecules. The atom with charge between − 0.2–0.2 is the hydrophobic area and the other is the hydrophilic area, the sum of these two parts should be equal to the total accessible surface area of solvent. With a certain total area, the larger the proportion of the hydrophobic area, the more important the hydrophobic interaction in the formation of aggregates or gel.

**Figure 8:**
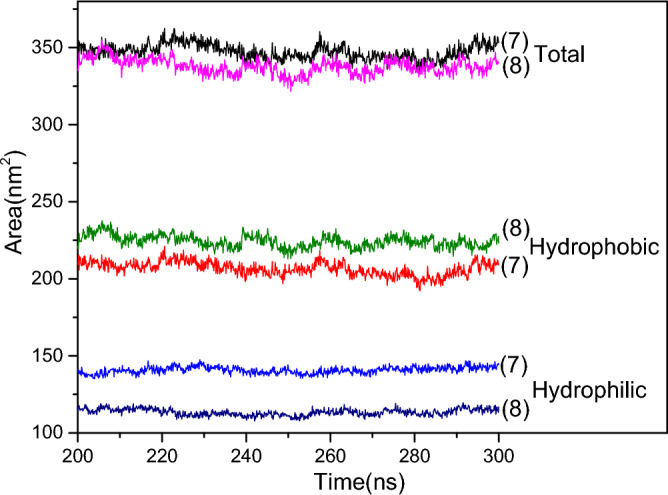
Solvent accessible surface area (sasa) of the group 2NapFF in systems (7) and (8). Including the total, hydrophobic, and hydrophilic sasa, for the 2NapFF.

Figure 8 shows the solvent-accessible surface area of the dipeptide group in systems (7) and (8) during the last 100 ns (from 200–300 ns) of the simulation process; that is, the aggregates are stabilized. It is clear that the total sasa of the dipeptide group in system (8) is slightly lower than that in system (7) at the same concentrations, indicating that the gel induced by Ca^2+^ in system (8) is more compact than the worm-like micelles induced by Na^+^ in system (7). The hydrophobic area in system (8) is clearly larger than that of system (7), and the hydrophilic area of system (7) is naturally higher than that of system (8), which indicates that the proportion of hydrophobic area in system (8) is significantly larger than that of system (7). Hence, the hydrophobic interaction plays an important role in the formation of the gel induced by Ca^2+^ in system (8).

## Conclusions

In this study, at high pH, molecular dynamics simulations were used to simulate the formation of aggregates triggered by Na and Ca ions in different concentrations of dipeptide (2NapFF) solutions, and it was found that the aggregation ability of dipeptide molecules triggered by high-valence Ca^2+^ was stronger than that of low-valence Na^+^. Simultaneously, it was confirmed that when the Na^+^ system formed a worm-like micelle, the Ca^2+^ system triggered gel formation.

The analysis of simulation results shows that the non-bond Van der Walls interaction, hydrophobic force, and electrostatic force, are the main driving forces for the formation of aggregations. The formation of gels in the dipeptide solutions triggered by Ca^2+^, wherein hydrogen bonds play no significant role in the aggregate formation. The electrostatic attraction drives Ca^2+^ to form weak coordination with four oxygen atoms on two carboxyl groups and the oxygen of surrounding water molecules, which makes it easier for dipeptide molecules to form a branched network structure gel rather than entanglement of worm-like micelles.

Therefore, it can be generalized that the weak coordination effect caused by electrostatic effect cannot be ignored in LMWG formation by small molecules of dipeptides triggered by salt ions, but the hydrophobic effect can be ignored.

## Simulation methods

### Tools for simulations

All simulations in this study were conducted by Gromacs 2022.1 package using the Optimized Potentials for Liquid Simulation all-atomic (OPLS-AA) method. The chemical structure of 2NapFF can be found in PubChem, the world’s open chemical database. The deprotonated structure was provided by Avogadro^39^ software, which removes H from its carboxyl group. The force field of OPLS-AA was provided by the web-based service LigParGen, with 1.14* CM1A particle atomic charges, and the particle mesh Ewald (PME)^40^ was used to calculate the long-range electrostatic interaction using Lennard–Jones (LJ). The electrostatic interactions were treated using the particle mesh Ewald (PME) technique. Visualizations of all molecular configurations were performed using the Molecular graphics software, VMD.

### Detail simulations

All simulations were conducted in the NpT ensemble with a pressure of p = 1 bar and temperature of 298 K using the Berendsen bath coupling scheme. The repulsive cutoff for the van der Waals term and the coulomb were chosen to be 1.2 nm, and the simple point charge (SPC) was chosen for water. Semi-isotropic pressure coupling was applied in OPLS-AA simulations. A Verlet leapfrog algorithm with a time step of 5 fs was used, and the trajectories were saved every 20 ps.

The compositions of all the simulation systems are listed in Table 1 (from (1) to (8), with eight systems in total). All systems had the same amount of solvent (14,000 water molecules) and different monomers (2NapFF) with the corresponding Na^+^ and Ca^2+^ ions to maintain the electrical neutrality of the solution in every simulation cell. To ensure that all aggregates were stable, the simulation time was increased to 300 ns for all cells. The odd systems (1, 3, 5, 7) are Na carboxylate systems, and even systems (2, 4, 6, 8) are Ca carboxylate systems.

## Data Availability

The datasets used and analyzed during the current study are available from the corresponding author upon reasonable request.
