# Author Correction: Molecular dynamics study of low molecular weight gel forming salt-triggered dipeptide

**DOI:** 10.1038/s41598-023-40759-5

**Published:** 2023-08-22

**Authors:** Xiangfeng Jia, Jingfei Chen, Wen Xu, Qi Wang, Xiaofeng Wei, Yongshan Ma, Feiyong Chen, Guiqin Zhang

**Affiliations:** 1 School of Municipal and Environmental Engineering, Shandong Jianzhu University, Jinan, 250101 Shandong China; 2 Key Laboratory Biofuels and Shandong Provincial Key Laboratory of Synthetic Biology, Qingdao Institute of Bioenergy and Bioprocess Technology, Chinese Academy of Sciences, Qingdao, 266101 China; 3 Institute of Resources and Environment Innovation, Shandong Jianzhu University, Jinan, 250101 Shandong China

Correction to: *Scientific Reports* https://doi.org/10.1038/s41598-023-33166-3, published online 18 April 2023

Feiyong Chen was omitted from the author list in the original version of this Article.

The original Article has been corrected.

